# Comparison of the anti-inflammatory effects of esomeprazole and fexuprazan in lipopolysaccharide-stimulated RAW 264.7 macrophages

**DOI:** 10.1186/s40360-026-01147-7

**Published:** 2026-05-09

**Authors:** Gi-Beom Ju, Seong Jun Kim, Daye Lee, Min Je Kim, Wan-Kyu Ko, Min Jai Cho, Seil Sohn

**Affiliations:** 1 Department of Life Science, CHA University, 335, Pangyo-ro, Bundang-gu, Seongnam-si, Gyeonggi-do 13488 Republic of Korea; 2 Department of Chemistry and Chemical Biology, Rutgers, The State University of New Jersey, 123 Bevier Road, Piscataway, NJ 08854 USA; 3 Department of Neurosurgery, Chungbuk National University College of Medicine, Chungbuk National University Hospital, 776, 1Sunhawn-ro, Seowon-gu, Cheongju- si, Chungcheong-do 28644 Republic of Korea; 4 Department of Neurosurgery, CHA Bundang Medical Center, 59, Yatap-ro, Bundang-gu, Seongnam-si, Gyeonggi-do 13496 Republic of Korea

**Keywords:** Esomeprazole, Fexuprazan, RAW 264.7, Lipopolysaccharide, Anti-inflammation, Voltage-gated potassium channel 1.3

## Abstract

**Background:**

This study aimed to examine the anti-inflammatory effects of esomeprazole (ESO) and fexuprazan (FEXU) in lipopolysaccharide (LPS)-stimulated RAW 264.7 macrophages and evaluated voltage-gated potassium channel 1.3 (Kv1.3) to infer the involved mechanism.

**Methods:**

The production of nitric oxide (NO) and the expression of pro-inflammatory mediators and Kv1.3, influenced by ESO and FEXU, were measured by means of a quantitative analysis of NO production and by a quantitative real-time polymerase chain reaction (qRT-PCR), respectively. In addition, we assessed the inhibitory effects of ESO and FEXU on the activation of the mitogen-activated protein kinase (MAPK) signaling pathway by examining the phosphorylation states of extracellular signal-regulated kinase (ERK), c-Jun N-terminal kinase (JNK), and p38, via a Western blot analysis. We also investigated the effects of ESO and FEXU on macrophage polarization by evaluating the expressions of inducible nitric oxide synthase (iNOS) and cluster of differentiation (CD) 206 through immunocytochemistry (ICC) staining.

**Results:**

The production of NO and the expression of pro-inflammatory mediators, as well as Kv1.3, were reduced by both ESO and FEXU. FEXU showed a greater inhibitory effect for pro-inflammatory mediators and Kv1.3. Both agents were found to suppress the activation of the MAPK signaling pathway, with FEXU reducing JNK and p38 phosphorylation and ESO reducing p38 phosphorylation. FEXU led to reduced iNOS expression with a consequent increase in CD206. In contrast, ESO did not induce significant changes in iNOS and CD206 expression levels.

**Conclusion:**

These results suggest that FEXU has a superior anti-inflammatory effect relative to that of ESO, indicating its potential as an anti-inflammatory agent.

**Supplementary Information:**

The online version contains supplementary material available at 10.1186/s40360-026-01147-7.

## Introduction

Inflammation serves as a fundamental defense mechanism when exposed to harmful agents such as pathogens, with macrophages playing a pivotal role by releasing cytokines that mediate the immune response [1]. In response to pathogen invasion and cellular stress, macrophages secrete pro-inflammatory mediators, including nitric oxide (NO), tumor necrosis factor-α (TNF-α), interleukin-1α (IL-1α), interleukin-1β (IL-1β), interleukin-6 (IL-6), and cyclooxygenase-2 (COX-2) [2–7].

Macrophage polarization exists along a functional spectrum rather than as a strict binary classification. Therefore, the terms M1-like and M2-like are employed to describe these phenotypes. M1-like macrophages release pro-inflammatory cytokines, whereas M2-like macrophages secrete anti-inflammatory cytokines [8]. The production of these inflammatory mediators is associated with the activation of the mitogen-activated protein kinase (MAPK) signaling pathway [9].

However, an overactive inflammatory response can result in tissue damage and pose a risk to human health [10]. Although current pharmacological treatments, such as corticosteroids and non-steroidal anti-inflammatory drugs (NSAIDs), are effective, they often have adverse side effects [11].

Esomeprazole (ESO), a proton-pump inhibitor (PPI), is used to treat gastroesophageal reflux disease (GERD) by covalently and irreversibly inhibiting H⁺/K⁺ ATPase in gastric parietal cells [12]. Recent studies suggest that ESO exerts additional therapeutic effects across various disease models, including sepsis, lung injury, renal ischemia–reperfusion injury, and hepatic fibrosis [13–16].

Fexuprazan (FEXU), a potassium-competitive acid blocker (PCAB), was developed to overcome the limitations of PPIs, such as its slow onset of action and dependence on acidic activation [17]. Unlike PPIs, FEXU inhibits H⁺/K⁺ ATPase in a reversible, K⁺-competitive, and pH-independent manner [17]. It is used in the treatment of acid-related disorders, including GERD and gastric ulcers. It has demonstrated comparable or superior efficacy to conventional PPIs in clinical studies [17].

The inflammatory activation of macrophages involves voltage-gated potassium channels, particularly voltage-gated potassium channel 1.3 (Kv1.3), which is critical for membrane potential regulation, cytokine production, macrophage migration, and activation [18].

According to a previous study, Kv1.3 plays an important role in macrophage activation and inflammatory responses [18]. Therefore, Kv1.3 expression was examined in this study to explore a potential association between ion channel regulation and the anti-inflammatory effects of ESO and FEXU. However, to the best of our knowledge, no studies have compared the anti-inflammatory effects of ESO and FEXU in macrophages. Hence, in this study, we aimed to assess the anti-inflammatory effects of ESO and FEXU in lipopolysaccharide (LPS)-stimulated RAW 264.7 macrophages.

## Materials and methods

### Preparation of ESO, FEXU, and LPS

ESO and FEXU were obtained from Daewoong Pharmaceutical Co., Ltd. (Seoul, Korea) and dissolved in dimethyl sulfoxide (DMSO, Thermo Fisher Scientific Inc., Waltham, MA, USA). Each experimental concentration of ESO and FEXU was prepared by diluting the samples in Dulbecco’s modified Eagle’s medium (DMEM, Gibco, MA, USA), which contained 10% fetal bovine serum (FBS, Gibco) and 1% penicillin-streptomycin (P/S, Gibco). LPS was purchased from Sigma Aldrich (St. Louis, MO, USA) and dissolved in Dulbecco’s phosphate-buffered saline (DPBS, Thermo) at a concentration of 1 mg/mL. To induce an inflammatory response in RAW 264.7 macrophages, it was diluted with DMEM to a concentration of 1 µg/mL.

### Cell culture

The RAW 264.7 murine macrophage cell line was purchased from the Korean Cell Lines Bank. It was cultured in DMEM with 10% FBS and 1% P/S at 37 °C in a 5% CO_2_ atmosphere. The cells were subcultured when they reached 90% confluence and were washed with DPBS, with fresh media replaced every two days.

### Cell viability assay

Cell viability was assessed at the following concentrations: ESO (100, 110, 120, and 130 µM), FEXU (10, 15, 20, and 30 µM), and at the highest concentration of DMSO, with or without LPS stimulation (1 µg/mL), using a cell viability kit (EZ-Cytox, Daeil Labservice, Seoul, Korea). RAW 264.7 macrophages were seeded into a 96-well plate (3 × 10^4^ cells/well) and treated with the indicated concentrations of ESO, FEXU, and DMSO for 24 h. After 24 h, a 10-fold diluted EZ-Cytox solution was added and left to react for 1 h. The resulting supernatant was measured by a microplate reader (Thermo) at a wavelength of 450 nm.

### Measurement of NO

The cells were seeded into a 96-well plate (1 × 10⁵ cells/well) and treated with different concentrations of ESO (20, 40, 60, 80, 100, 110, and 120 µM) or FEXU (10, 15, and 20 µM), with or without LPS (1 µg/mL) for 24 h. In LPS-treated groups, ESO or FEXU was added simultaneously with LPS stimulation. The NO that accumulated in the supernatant was detected using a Griess Reagent Kit (Invitrogen, Carlsbad, CA, USA). Briefly, an equal volume of sulfanilamide was mixed with the supernatant. The mixture was left to incubate at room temperature for 30 min. Thereafter, a microplate reader was used to measure the absorbance of each group at a wavelength of 548 nm. The concentration of NO in the supernatant was determined based on a standard curve prepared from sodium nitrite.

### Experimental group

The differences among six groups were compared: a control (Ctrl) group without any treatment, a group with the 120 µM ESO treatment only, a group with the 20 µM FEXU treatment only, a group with the LPS treatment only, a group with the LPS and 120 µM ESO treatments, and a group with the LPS and 20 µM FEXU treatments.

### qRT-PCR

RAW 264.7 macrophages (2 × 10^6^ cells/well) were seeded into a 6-well plate for qRT-PCR analysis. The cells were treated with ESO (120 µM) or FEXU (20 µM), with or without LPS (1 µg/mL). In LPS-treated groups, ESO or FEXU was added simultaneously with LPS stimulation. After 6 h, the total RNA of the seeded cells was extracted using the TRIzol reagent (Invitrogen), following the manufacturer’s instructions. The qRT-PCR assessment was conducted as previously described [19]. The primers were obtained from Bioneer (Daejeon, Korea). Relative gene expression values for TNF-α, IL-1α, IL-1β, IL-6, COX-2, and Kv1.3 were normalized to those for glyceraldehyde 3-phosphate dehydrogenase (GAPDH) using the 2-ΔΔCT calculation. The nucleotide sequences of the primers are listed in Table 1.

**Table 1:** Nucleotide sequences of primers used in qRT-PCR

Gene	Forward (5’ → 3’)	Reverse (5’ → 3’)
TNF-α	TTGACCTCAGCGCTGAGTTG	CCTGTAGCCCACGTCGTAGC
IL-1α	TTGGTTAAATGACCTGCAACA	GAGCGCTCACGAACAGTTG
IL-1β	TGCAGAGTTCCCCAACTGGTACATC	GTGCTGCCTAATGTCCCCTTGAATC
IL-6	GCTACCAAACTGGATATAATCAGGA	CCAGGTAGCTATGGTACTCCAGAA
COX-2	CGGAGGAGAAGTGGGGTTTAGGAT	TGGGAGGCACTTGCGTTGATGG
Kv1.3	AGTATATGGTGATCGAAGAGG	AGTGAATATCTTCTTGATGTT
GAPDH	CTCATGACCACAGTCCATGC	TTCATCGGGATGACCTT

### Western blot

RAW 264.7 macrophages (2 × 10^6^ cells/dish) were seeded into a 100 × 20 mm culture dish. The cells were treated with ESO (120 µM) or FEXU (20 µM), with or without LPS (1 µg/mL). In LPS-treated groups, ESO or FEXU was added simultaneously with LPS stimulation. After 30 min and 24 h, the cells were gently scraped off the plate using a cell scraper and collected in 1.5 mL tubes. The cells were lysed by adding RIPA lysis buffer (Sigma) containing a protease inhibitor (Roche Diagnostics, Mannheim, Germany) and a phosphatase inhibitor cocktail (Sigma), with the mixture then incubated on ice for 30 min, after which it was centrifuged at 13,000 rpm for 30 min. The concentration of the isolated protein was measured using a microplate reader at 562 nm. Western blotting was conducted as described previously [20]. The volumes of the phosphorylated forms of extracellular signal-regulated kinase (p-ERK, 1:1000), c-Jun N-terminal kinase (p-JNK, 1:1000), and p38 (p-p38, 1:1000), as well as the total forms of ERK (t-ERK, 1:1000), JNK (t-JNK, 1:1000), and p38 (t-p38, 1:1000), were quantified. ERK, JNK, and p38 antibodies in p/t form were purchased from Cell Signaling Technology (Danvers, MA, USA), and β-actin antibody was purchased from Abcam (Cambridge, UK). As an internal control, the membranes were probed for β-actin (1:5000). The membranes were incubated with primary antibodies overnight at 4℃ and were treated with a secondary antibody (1:5000; GeneTex, Irvine, CA, USA) for 2 h at room temperature. ECL solution (Amersham Biosciences, Piscataway, NJ, USA) was used to detect the visualized signal bands via a luminescent image analyzer (Fujifilm, Tokyo, Japan). The volumes of phosphorylated form/total form (p/t form) were calculated and quantified using ImageJ software.

### ICC staining

RAW 264.7 macrophages (4 × 10^4^ cells/well) were seeded into a 6-well glass-bottom plate. The cells were treated with ESO (120 µM) or FEXU (20 µM), with or without LPS (1 µg/mL). In LPS-treated groups, ESO or FEXU was added simultaneously with LPS stimulation. After 24 h, the culture media were removed, and the cells were fixed with 4% paraformaldehyde (PFA) for 10 min. Afterwards, Triton X-100 was used to permeabilize the cells for 5 min, after which 2% bovine serum albumin (BSA) was used to block them for 1 h. Thereafter, the cells were treated with an anti-inducible nitric oxide synthase (iNOS) antibody (1:50, Abcam) and the anti-mouse macrophage mannose receptor (MMR)/cluster of differentiation (CD) 206 antibody (1:100, R&D Systems, Minneapolis, MN, USA) at 4℃ overnight. Subsequently, Alexa 568-conjugated (1:400, Invitrogen) and Alexa 647-conjugated (1:800, Invitrogen) secondary antibodies were used to stain the cells for 1 h at room temperature. After washing the remaining secondary antibodies, the cells were mounted in VECTASHIELD Vibrance Antifade Medium with DAPI (Vector Laboratories, Burlingame, CA, USA). A Zeiss LSM 880 confocal microscope was used to detect the fluorescent intensity of Immunocytochemistry (ICC) staining. The mean fluorescence intensity of iNOS or CD206 was measured from randomly selected fields under identical imaging conditions and normalized to the number of DAPI-positive nuclei to account for differences in cell density.

### Statistical analysis

All values are presented as the mean ± standard deviation (SD). A one-way analysis of variance (ANOVA) followed by a Tukey’s post hoc test was used to verify and compare statistical differences between the groups. Differences in *p*-values of * *p* < 0.05, ** *p* < 0.01, and *** *p* < 0.001 were considered statistically significant.

## Results

### Cytotoxicity of ESO and FEXU on RAW 264.7 macrophages

**Fig. 1:**
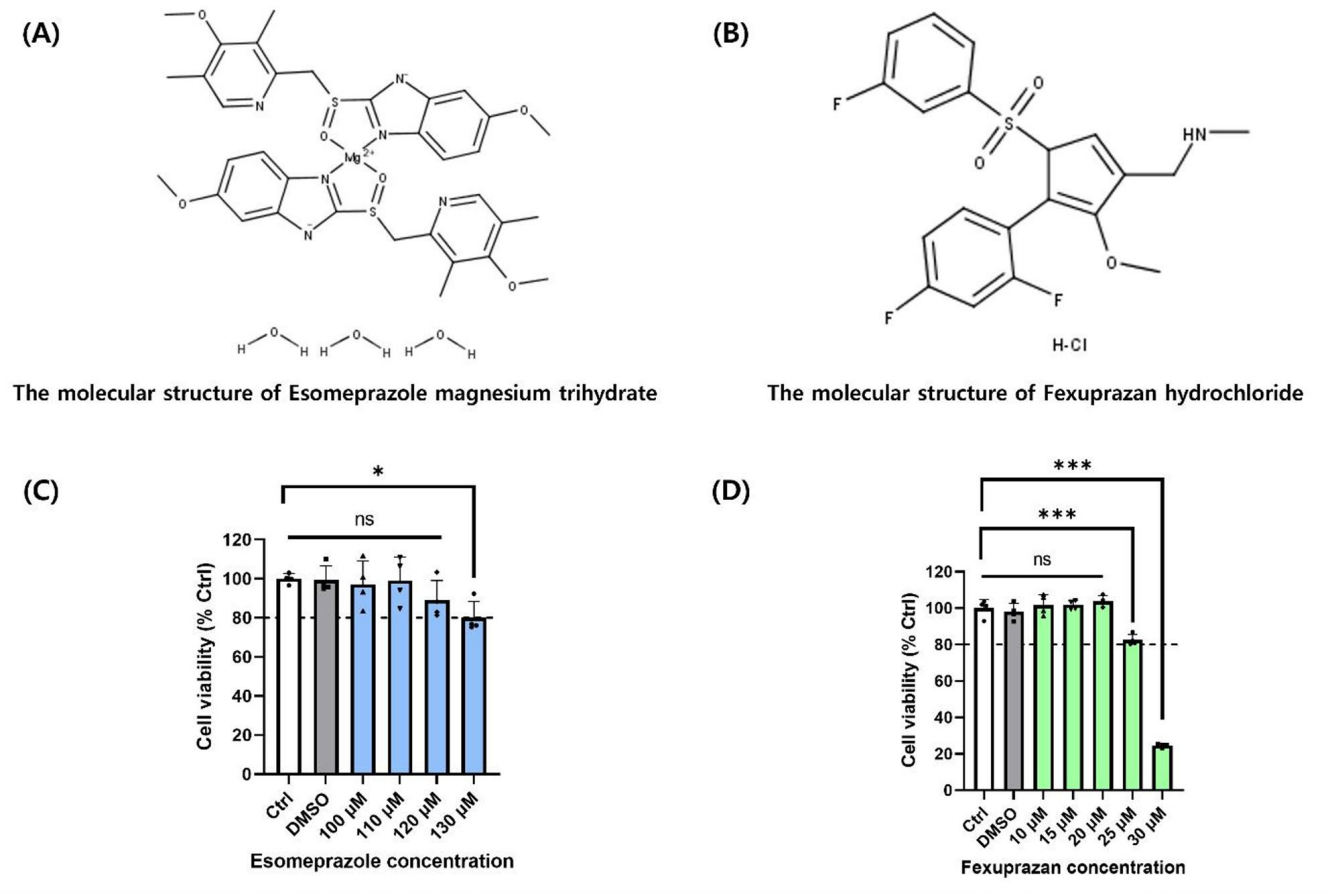
The molecular structures of esomeprazole magnesium trihydrate (ESO, **A**) and fexuprazan hydrochloride (FEXU, **B**), and their effects on RAW 264.7 cell viability (**C** and **D**). ESO (100, 110, 120, and 130 µM) and FEXU (10, 15, 20, 25, and 30 µM) were treated for 24 h. The results are presented as the mean ± standard deviation (SD, *n* = 4 per group), not significant (ns), * *p* < 0.05, and *** *p* < 0.001

Figure 1A and B present the molecular structure of ESO and FEXU. The cytotoxicity levels of ESO and FEXU were assessed. ESO doses ranging from 100 µM to 120 µM, FEXU doses ranging from 10 µM to 20 µM, and the group treated only with DMSO, in amounts equal to those of the highest concentration group of ESO and FEXU, did not significantly increase or decrease cell viability (Fig. 1C and D, not significant: ns). However, a significant decline was observed at 130 µM of ESO and 25 and 30 µM of FEXU (Fig. 1C and D, * *p* < 0.05 and *** *p* < 0.001). Additional cell viability assays performed under LPS-stimulated conditions confirmed that ESO and FEXU did not significantly affect cell viability at the concentrations used in this study (Fig. S1).

### Inhibitory effect of NO production by ESO and FEXU

**Fig. 2:**
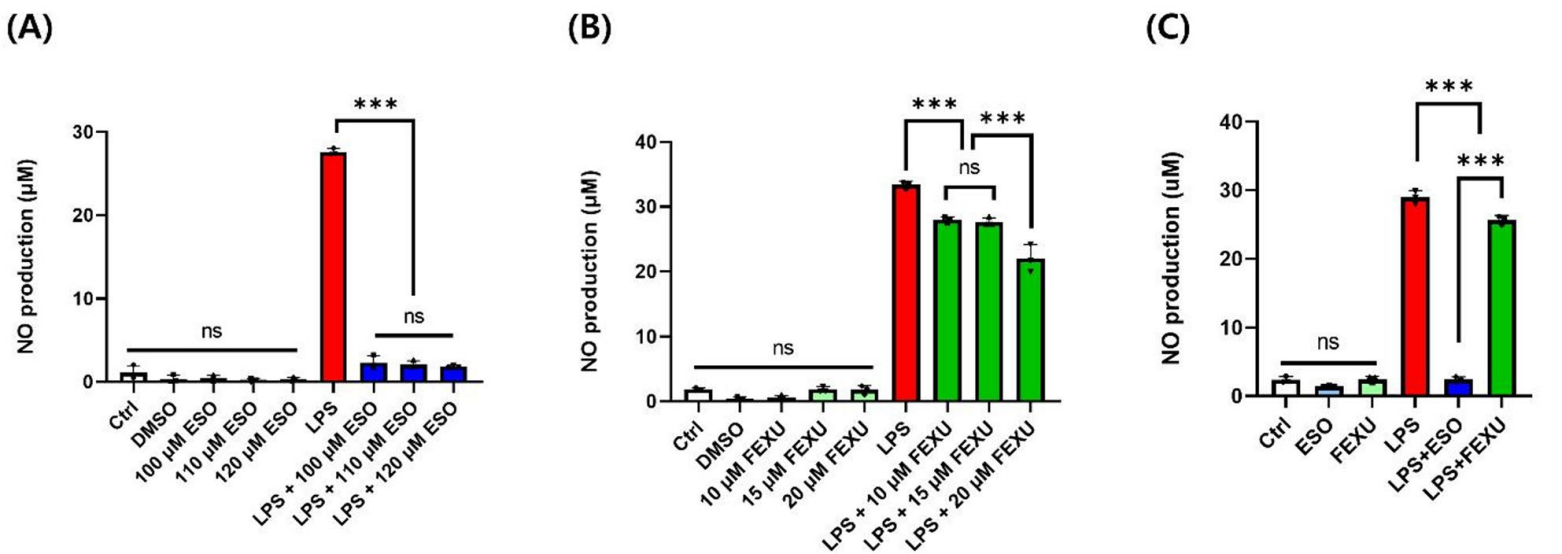
Inhibitory effect on nitric oxide (NO) production by ESO and FEXU in lipopolysaccharide (LPS)-stimulated RAW 264.7 macrophages. Macrophages were treated with LPS (1 µg/mL), ESO (100, 110, and 120 µM), and FEXU (10, 15, and 20 µM) for 24 h. The results are presented as the mean ± SD (*n* = 3 per group), ns, and *** *p* < 0.001

To evaluate whether ESO and FEXU inhibit NO production, we quantified NO production in LPS-stimulated RAW 264.7 macrophages. First, we assessed the concentrations of the ESO samples (20, 40, 60, 80, 100, 110, and 120 µM), the FEXU samples (10, 15, and 20 µM), and the group treated with DMSO in amounts equal to those of the highest concentration group of ESO and FEXU. ESO and FEXU without LPS and the DMSO group did not induce any significant increase in NO production (Fig. 2A and B, ns). However, a significant increase in NO production was observed in the LPS group (Fig. 2A and B, *** *p* < 0.001). Although the effect was not strictly dose-dependent, ESO treatment groups significantly reduced this increase in NO production (Fig. 2A, ns and *** *p* < 0.001). In contrast, FEXU treatment groups led to a significant, dose-dependent reduction in NO production (Fig. 2B, ns and *** *p* < 0.001). Afterwards, we compared the highest concentrations of ESO and FEXU in Fig. 2A and B (Fig. 2C, *** *p* < 0.001). Additional experiments using lower concentrations of ESO showed only modest inhibitory effects on NO production compared with the concentrations used in Fig. 2A (Fig. S2).

### ESO and FEXU suppressed pro-inflammatory mediators in LPS-stimulated RAW 264.7 macrophages

**Fig. 3:**
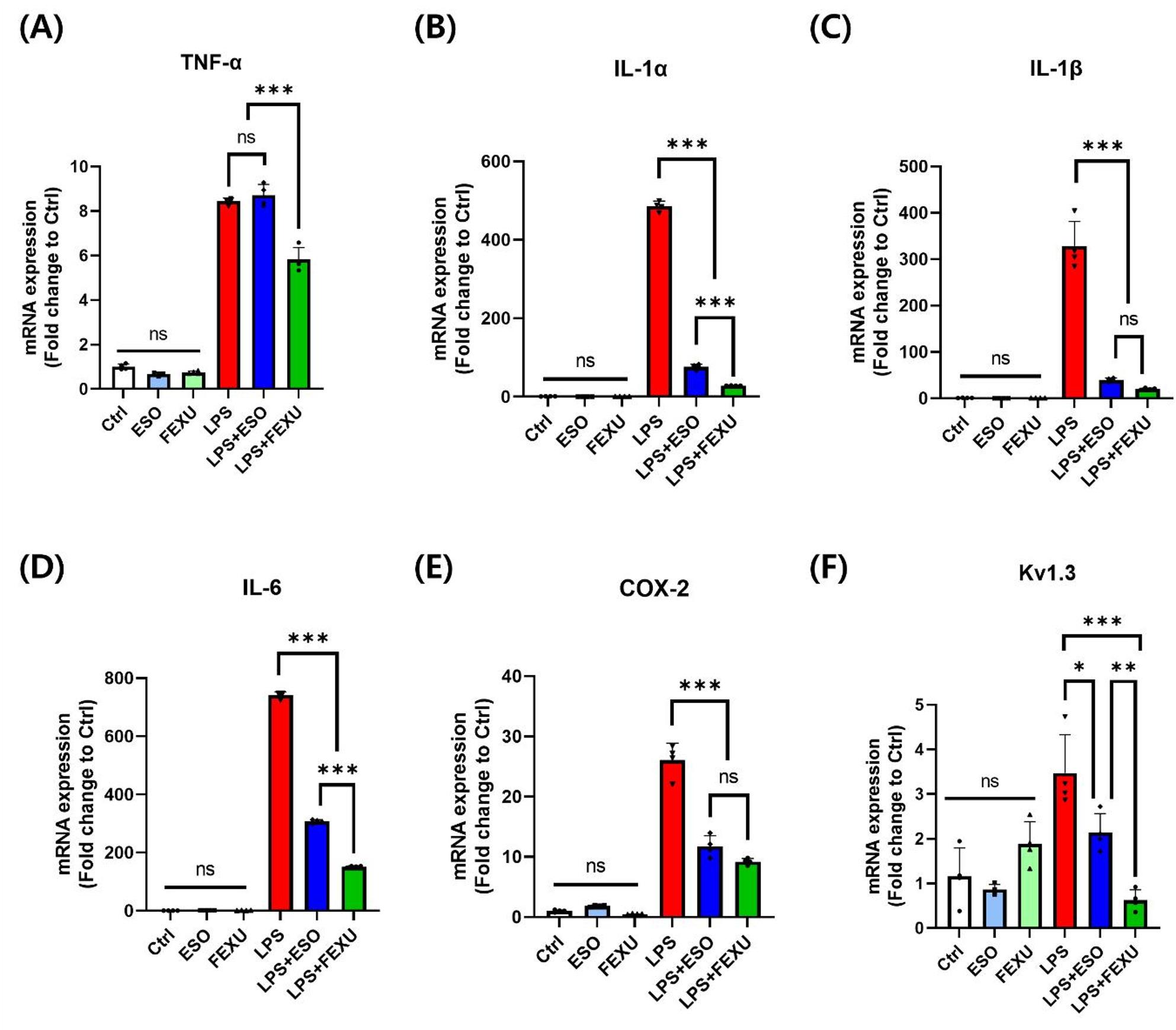
The mRNA expressions of pro-inflammatory mediators, tumor necrosis factor-α (TNF-α, **A**), interleukin-1α (IL-1α, **B**), interleukin-1β (IL-1β, **C**), interleukin-6 (IL-6, **D**), and cyclooxygenase-2 (COX-2, **E**), as well as voltage-gated potassium channel 1.3 (Kv1.3, **F**), were examined in RAW 264.7 macrophages treated with ESO (120 µM) or FEXU (20 µM) in the presence of LPS (1 µg/mL) for 6 h. The results are presented as the mean ± SD (*n* = 4 per group), ns, * *p* < 0.05, ** *p* < 0.01, and *** *p* < 0.001

To investigate whether ESO and FEXU modulate mRNA expression of pro-inflammatory mediators in LPS-stimulated RAW 264.7 macrophages, qRT-PCR was conducted after 6 h of LPS stimulation. ESO and FEXU alone did not induce the mRNA expression of pro-inflammatory mediators or Kv1.3 (Fig. 3A-F, ns). The mRNA levels of TNF-α, IL-1α, IL-1β, IL-6, COX-2 and Kv1.3 were markedly elevated following the LPS treatment (Fig. 3A-F, *** *p* < 0.001). Co-treatment with FEXU significantly attenuated the expression levels of these pro-inflammatory mediators, with comparable or greater efficacy than ESO in most cases (Fig. 3A-E, ns and *** *p* < 0.001). Notably, the mRNA levels of Kv1.3 were also reduced by ESO and FEXU, with FEXU producing a greater reduction (Fig. 3F, * *p* < 0.05, ** *p* < 0.01, and *** *p* < 0.001).

### Effect of ESO and FEXU on the phosphorylation of ERK, JNK, and p38 in the MAPK signaling pathway in LPS-stimulated RAW 264.7 macrophages

**Fig. 4:**
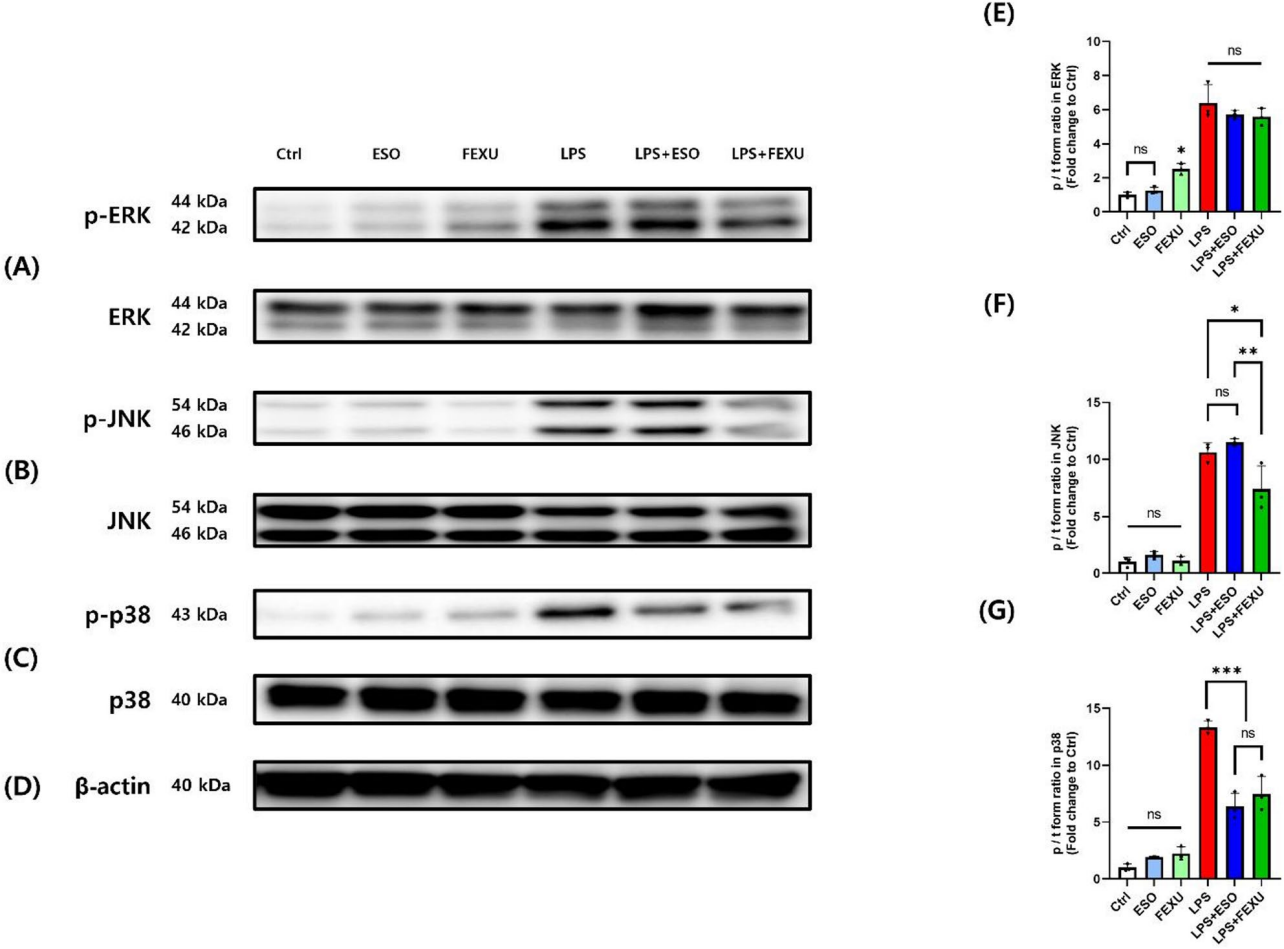
The phosphorylation activities of the mitogen-activated protein kinase (MAPK) signaling pathway in LPS (1 µg/mL)-stimulated RAW 264.7 macrophages treated with ESO (120 µM) or FEXU (20 µM) after 30 min. Representative images of the p and t forms of extracellular signal-regulated kinase (ERK, **A**), c-Jun N-terminal kinase (JNK, **B**), p38 (**C**), and β-actin (**D**). Quantitative analysis of the p / t ratios of ERK (**E**), JNK (**F**), and p38 (**G**). The results are presented as the mean ± SD (*n* = 3 per group), ns, * *p* < 0.05, ** *p* < 0.01, and *** *p* < 0.001

To determine the effects of ESO and FEXU on the MAPK signaling pathway, the phosphorylation levels of ERK, JNK, and p38 were measured by Western blotting after 30 min of LPS treatment in RAW 264.7 macrophages (Fig. 4A-G). At this time point, LPS significantly increased the phosphorylation of ERK, JNK, and p38 (Fig. 4E-G, *** *p* < 0.001). Co-treatment with FEXU reduced the phosphorylation of JNK (Fig. 4F, * *p* < 0.05) and p38 (Fig. 4G, *** *p* < 0.001), whereas no significant reduction was observed in ERK phosphorylation (Fig. 4E, ns). In contrast, co-treatment with ESO reduced the phosphorylation of p38 (Fig. 4G, *** *p* < 0.001), whereas no significant reduction was observed in ERK and JNK phosphorylation (Fig. 4E and F, ns). At 24 h of treatment, LPS significantly increased the phosphorylation of ERK and JNK (Fig. S3E and F, *** *p* < 0.001), whereas phosphorylation of p38 was not significantly increased (Fig. S3F, ns). Co-treatment with ESO and FEXU reduced the phosphorylation of ERK (Fig. S3E, * *p* < 0.05 and *** *p* < 0.001) and JNK (Fig. S3F, *** *p* < 0.001). However, no significant suppressive effect of ESO or FEXU was observed in the p38 (Fig. S3G, ns). Interestingly, treatment with FEXU alone slightly increased the phosphorylation of p38 compared with the Ctrl group (Fig. S3G, * *p* < 0.05).

### FEXU increases differentiation in M2-like phenotype macrophages

**Fig. 5:**
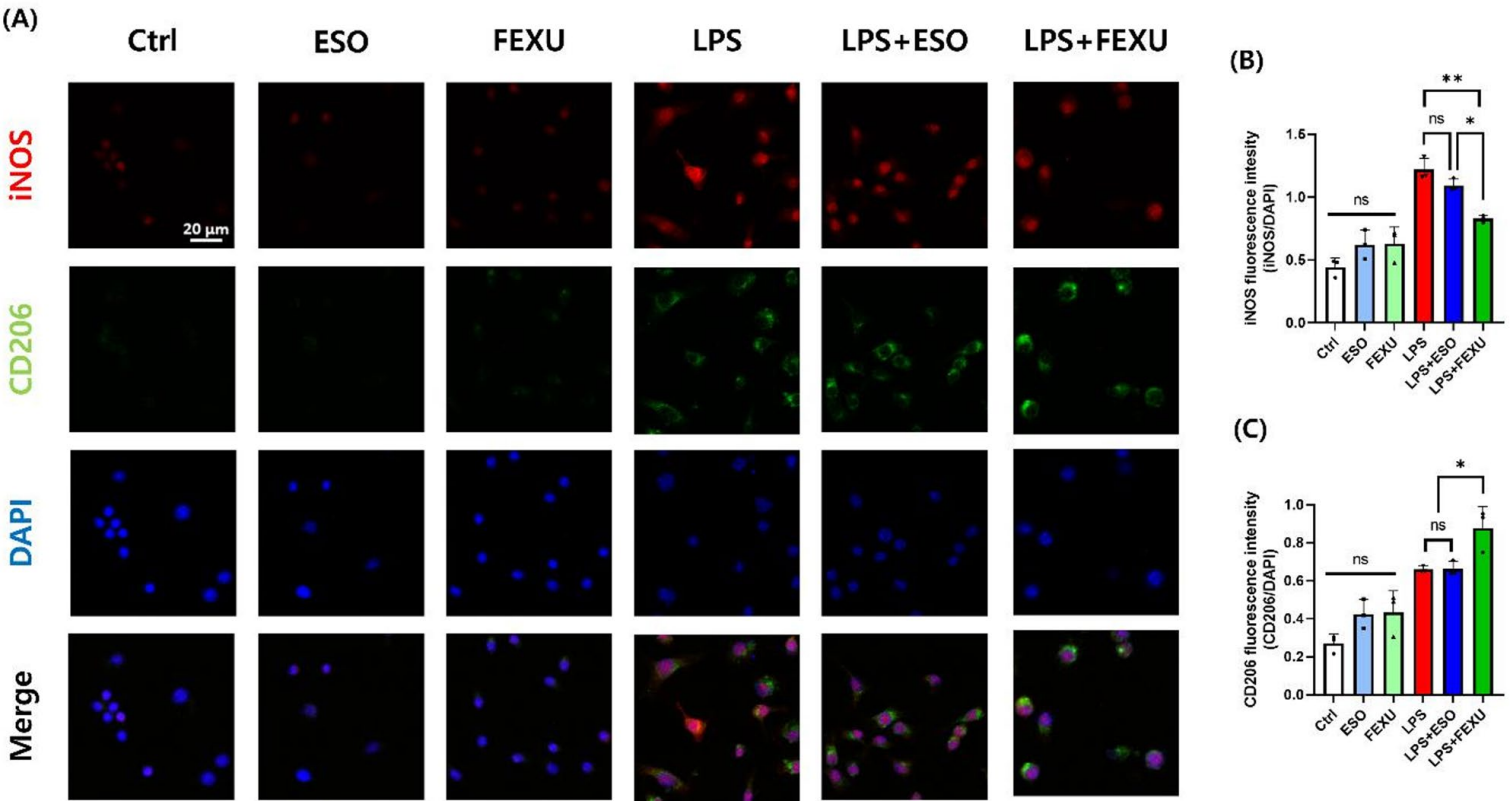
Inducible nitric oxide (iNOS) and cluster of differentiation (CD) 206 expressions in LPS (1 µg/mL)-stimulated RAW 264.7 macrophages treated with ESO (120 µM) or FEXU (20 µM) were measured after 24 h (**A**, scale bar = 20 μm). Quantitative analysis of the iNOS fluorescence intensity levels (**B**) and CD206 fluorescence intensity levels (**C**). The results are presented as the mean ± SD (*n* = 3 per group), ns, * *p* < 0.05, and ** *p* < 0.01

To determine whether ESO or FEXU upregulates M2-like differentiation, ICC staining was conducted after 24 h of LPS stimulation. iNOS is commonly used as a marker of M1-like macrophages, whereas CD206 is associated with M2-like macrophage polarization [21]. As shown in Fig. 5B, ESO and FEXU alone did not induce iNOS expression (Fig. 5B, ns). The expression of iNOS was significantly increased following the LPS treatment (Fig. 5B, *** *p* < 0.001). Co-treatment with FEXU significantly reduced the expression of iNOS, whereas co-treatment with ESO showed no significant change (Fig. 5B, ns, * *p* < 0.05, and ** *p* < 0.01). Furthermore, co-treatment with FEXU significantly increased the expression of CD206 compared to the LPS group, while co-treatment with ESO showed no significant change (Fig. 5C, ns and * *p* < 0.05).

## Discussion

ESO, a PPI that irreversibly inhibits gastric H⁺/K⁺ ATPase via covalent binding, has been used to treat GERD [12]. A recent study demonstrated that ESO shows anti-inflammatory effects by reducing TNF-α and IL-1β in human monocytes [13] and reducing iNOS in lung epithelial cells [14].

FEXU, a PCAB that inhibits gastric H^+^ / K^+^ ATPase in a reversible and K^+^-competitive manner, was developed to overcome limitations of PPI [17]. Recently, it was reported that FEXU shows an anti-inflammatory effect by reducing pro-inflammatory cytokines and suppressing NLRP1/Caspase-1/GSDMD pyroptotic pathway in human esophageal cells and human acute monocytic leukemia THP-1 cells [22].

In this study, we compared the anti-inflammatory effects of ESO and FEXU in LPS-stimulated RAW 264.7 macrophages. First, to determine the cytotoxic concentration of ESO and FEXU, RAW 264.7 macrophages were treated with different concentrations of ESO (100, 110, 120, and 130 µM) and FEXU (10, 15, 20, 25, and 30 µM). Cell viability exceeding 80% is considered a non-toxic density level [23], and we found that ESO did not demonstrate cytotoxicity up to 120 µM and that FEXU demonstrated no cytotoxicity up to 20 µM (Fig. 1C and D).

NO, a key signaling molecule, acts as a pro-inflammatory mediator whose excessive production under pathological conditions contributes to the development of inflammation [24]. To find the optimal concentration that exhibits an anti-inflammatory effect, production levels of NO were compared. ESO showed the greatest decrease in NO production at 120 µM, and FEXU showed the greatest decrease at 20 µM (Fig. 2A and B). Subsequent experiments were conducted with these concentrations.

To evaluate the anti-inflammatory effects of ESO and FEXU, the mRNA expression levels of Kv1.3 and pro-inflammatory mediators, in this case TNF-α, IL-1α, IL-1β, IL-6, and COX-2, were compared using qRT-PCR. FEXU exerted comparable or superior inhibitory effects on pro-inflammatory mediators compared to ESO (Fig. 3A-E). Notably, FEXU significantly reduced the mRNA expression of all five pro-inflammatory mediators tested, whereas ESO showed only partial suppression, with no significant effect on TNF-α.

A previous study has suggested that modulation of Kv1.3 activity can influence macrophage polarization and inflammatory signaling [18]. Therefore, Kv1.3 expression was evaluated in this study to explore a potential association between ion channel regulation and macrophage inflammatory responses. In the present study, Kv1.3 mRNA expression was significantly reduced by both ESO and FEXU under LPS-stimulated conditions, with FEXU showing a greater reduction (Fig. 3F). However, since Kv1.3 was assessed only at the mRNA level, the current results do not establish a direct mechanistic role. Further studies, including protein-level and functional analysis, will be required to clarify whether Kv1.3 directly contributes to the anti-inflammatory effects of these compounds. These findings suggest that ESO and FEXU exert anti-inflammatory effects in macrophages, while the potential involvement of Kv1.3 warrants further investigation.

The MAPK signaling pathway is often activated through phosphorylation. The activated MAPK signaling pathway is involved in cellular functions, including inflammation by releasing pro-inflammatory cytokines [9]. In the present study, MAPK phosphorylation was analyzed at 30 min and 24 h after LPS stimulation. At 30 min, LPS induced the phosphorylation of ERK, JNK, and p38. In contrast, at 24 h, LPS induced the phosphorylation of ERK and JNK, whereas p38 phosphorylation was not significantly increased. Under these conditions, ESO reduced p38 phosphorylation at 30 min, and the phosphorylation of ERK and JNK at 24 h. Similarly, FEXU reduced the JNK and p38 phosphorylation at 30 min, and the phosphorylation of ERK and JNK at 24 h. Both ESO and FEXU inhibited p38 phosphorylation at the early time point. In contrast, at the later time point, both agents inhibited ERK and JNK phosphorylation. These findings suggest that the effects of ESO and FEXU on the MAPK signaling pathway may be time-dependent. Hence, further studies are required to clarify the underlying mechanism.

In addition to the MAPK signaling pathway, the NF-κB pathway plays a central role in LPS-induced inflammatory responses by regulating the transcription of pro-inflammatory cytokines such as TNF-α, IL-1α, IL-1β, and IL-6 [25]. The observed anti-inflammatory effects of ESO and FEXU may also involve modulation of NF-κB signaling or other upstream pathways such as TLR4-mediated signaling [26]. Further studies are required to clarify the upstream regulatory mechanisms underlying these effects.

In the context of macrophage polarization, iNOS is a key enzyme primarily associated with the M1-like phenotype, and CD206 is associated with the M2-like phenotype [21]. FEXU significantly reduced the expression of iNOS, whereas ESO showed no significant change (Fig. 5A and B). Notably, CD206 was exclusively upregulated in response to a FEXU treatment (Fig. 5A and C). These results suggest that FEXU promotes an anti-inflammatory M2-like phenotype more effectively than ESO.

Taken together, our results demonstrate that both ESO and FEXU treatment reduced Kv1.3 mRNA expression in parallel with pro-inflammatory markers in LPS-stimulated RAW 264.7 macrophages, suggesting a potential association that warrants further mechanistic investigation.

## Conclusion

Both ESO and FEXU exhibited anti-inflammatory effects in LPS-stimulated RAW 264.7 macrophages. FEXU demonstrated more prominent reductions in the expression levels of pro-inflammatory mediators compared to ESO and promoted M2-like macrophage polarization. Kv1.3 mRNA expression was also reduced under these conditions. These findings suggest that FEXU, a PCAB, may represent a potential anti-inflammatory agent.

## Supplementary Information

Below is the link to the electronic supplementary material.

Supplementary Material 1

Supplementary Material 2

Supplementary Material 3

Supplementary Material 4

Supplementary Material 5

Supplementary Material 6

## Data Availability

Data will be made available on request.

## Abbreviations

ESO: Esomeprazole
FEXU: Fexuprazan
LPS: Lipopolysaccharide
Kv1.3: Voltage-gated potassium channel 1.3
NO: Nitric oxide
qRT-PCR: Quantitative real-time polymerase chain reaction
MAPK: Mitogen-activated protein kinase
ERK: Extracellular signal-regulated kinase
JNK: c-Jun N-terminal kinase
iNOS: Nitric oxide synthase
CD: Cluster of differentiation
ICC: Immunocytochemistry
TNF-α: Tumor necrosis factor-α
IL-1α: Interleukin-1α
IL-1β: Interleukin-1β
IL-6: Interleukin-6
COX-2: Cyclooxygenase-2
NSAIDs: Non-steroidal anti-inflammatory drugs
GERD: Gastroesophageal reflux disease
PPI: Proton-pump inhibitor
PCAB: Potassium-competitive acid blocker
DMSO: Dimethyl sulfoxide
DMEM: Dulbecco’s modified Eagle’s medium
FBS: Fetal bovine serum
P/S: Penicillin-streptomycin
DPBS: Dulbecco’s phosphate-buffered saline
Ctrl: Control
PFA: Paraformaldehyde
BSA: Bovine serum albumin
MMR: Macrophage mannose receptor
SD: Standard deviation
ANOVA: Analysis of variance

## References

[1] Medzhitov R. Origin and physiological roles of inflammation. Nature. 2008;454:428–35. 18650913 10.1038/nature07201

[2] MacMicking J, Xie Q-w, Nathan C. Nitric oxide and macrophage function. Annu Rev Immunol. 1997;15:323–50. 9143691 10.1146/annurev.immunol.15.1.323

[3] Parameswaran N, Patial S. Tumor necrosis factor-α signaling in macrophages. Crit Reviews™ Eukaryot Gene Expression. 2010;20. 10.1615/critreveukargeneexpr.v20.i2.10 PMC3066460 21133840

[4] Di Paolo NC, Shayakhmetov DM. Interleukin 1α and the inflammatory process. Nat Immunol. 2016;17:906–13. 27434011 10.1038/ni.3503 PMC5152572

[5] Dinarello CA. Immunological and inflammatory functions of the interleukin-1 family. Annu Rev Immunol. 2009;27:519–50. 19302047 10.1146/annurev.immunol.021908.132612

[6] Hirano T. IL-6 in inflammation, autoimmunity and cancer. Int Immunol. 2021;33:127–48. 33337480 10.1093/intimm/dxaa078 PMC7799025

[7] Nathan C. Points of control in inflammation. Nature. 2002;420:846–52. 12490957 10.1038/nature01320

[8] Yunna C, Mengru H, Lei W, Weidong C. Macrophage M1/M2 polarization. Eur J Pharmacol. 2020;877:173090. 32234529 10.1016/j.ejphar.2020.173090

[9] Cargnello M, Roux PP. Activation and function of the MAPKs and their substrates, the MAPK-activated protein kinases. Microbiol Mol Biol Rev. 2011;75:50–83. 21372320 10.1128/MMBR.00031-10 PMC3063353

[10] Rajendran P, Chen YF, Chen YF, Chung LC, Tamilselvi S, Shen CY, Day CH, Chen RJ, Viswanadha VP, Kuo WW. The multifaceted link between inflammation and human diseases. J Cell Physiol. 2018;233:6458–71. 29323719 10.1002/jcp.26479 PMC13482531

[11] Paglia MDG, Silva MT, Lopes LC, Barberato-Filho S, Mazzei LG, Abe FC, de Cássia Bergamaschi C. Use of corticoids and non-steroidal anti-inflammatories in the treatment of rheumatoid arthritis: Systematic review and network meta-analysis. PLoS ONE. 2021;16:e0248866. 33826610 10.1371/journal.pone.0248866 PMC8026036

[12] Kalaitzakis E, Björnsson E. A review of esomeprazole in the treatment of gastroesophageal reflux disease (GERD). Therapeutics and clinical risk management. 2007;3:653–663. PMC2374928 18472988

[13] Balza E, Piccioli P, Carta S, Lavieri R, Gattorno M, Semino C, Castellani P, Rubartelli A. Proton pump inhibitors protect mice from acute systemic inflammation and induce long-term cross-tolerance. Cell Death Dis. 2016;7:e2304–2304. 27441656 10.1038/cddis.2016.218 PMC4973356

[14] Ebrahimpour A, Wang M, Li L, Jegga AG, Bonnen MD, Eissa NT, Raghu G, Jyothula S, Kheradmand F, Hanania NA. Esomeprazole attenuates inflammatory and fibrotic response in lung cells through the MAPK/Nrf2/HO1 pathway. J Inflamm. 2021;18:17. 10.1186/s12950-021-00284-6 PMC8136131 34011367

[15] Ozkan TA, Karakoyunlu N, Polat R, Sarıbaş GS, Şener NC, Özdemir S, Peker K, Ünal D, Tuygun C. An evaluation of the protective effect of esomeprazole in an experimental model of renal ischemia–reperfusion. Int Urol Nephrol. 2018;50:217–23. 29280047 10.1007/s11255-017-1775-8

[16] Eltahir HM, Nazmy MH. Esomeprazole ameliorates CCl4 induced liver fibrosis in rats via modulating oxidative stress, inflammatory, fibrogenic and apoptotic markers. Biomed Pharmacother. 2018;97:1356–65. 29156525 10.1016/j.biopha.2017.11.028

[17] Ramani A, Merchant A, Cash BD. Review of the clinical development of fexuprazan for gastroesophageal reflux–related disease. Eur J Clin Pharmacol. 2023;79:1023–9. 37344679 10.1007/s00228-023-03521-4

[18] Man Q, Gao Z, Chen K. Functional potassium channels in macrophages. J Membr Biol. 2023;256:175–87. 36622407 10.1007/s00232-022-00276-4

[19] Ko W-K, Lee S-H, Kim SJ, Jo M-J, Kumar H, Han I-B, Sohn S. Anti-inflammatory effects of ursodeoxycholic acid by lipopolysaccharide-stimulated inflammatory responses in RAW 264.7 macrophages. PLoS ONE. 2017;12:e0180673. 28665991 10.1371/journal.pone.0180673 PMC5493427

[20] Kim S-J, Ko W-K, Han G-H, Lee D, Lee Y, Sheen S-H, Hong J-B, Sohn S. Chirality-Dependent Anti-Inflammatory Effect of Glutathione after Spinal Cord Injury in an Animal Model. Pharmaceuticals. 2021;14:792. 34451889 10.3390/ph14080792 PMC8398565

[21] Feito MJ, Diez-Orejas R, Cicuéndez M, Casarrubios L, Rojo JM, Portolés MT. Characterization of M1 and M2 polarization phenotypes in peritoneal macrophages after treatment with graphene oxide nanosheets. Colloids Surf B. 2019;176:96–105. 10.1016/j.colsurfb.2018.12.063 30594708

[22] Kim SY, Yoon J-H, Jung DH, Kim GH, Kim CH, Lee SK. Fexuprazan safeguards the esophagus from hydrochloric acid-induced damage by suppressing NLRP1/Caspase-1/GSDMD pyroptotic pathway. Front Immunol. 2024;15:1410904. 39737189 10.3389/fimmu.2024.1410904 PMC11682960

[23] Iwasawa A, Ayaki M, Niwano Y. Cell viability score (CVS) as a good indicator of critical concentration of benzalkonium chloride for toxicity in cultured ocular surface cell lines. Regul Toxicol Pharmacol. 2013;66:177–83. 23557985 10.1016/j.yrtph.2013.03.014

[24] Sharma J, Al-Omran A, Parvathy S. Role of nitric oxide in inflammatory diseases. Inflammopharmacology. 2007;15:252–9. 18236016 10.1007/s10787-007-0013-x

[25] Liu T, Zhang L, Joo D, Sun S-C. NF-κB signaling in inflammation. Signal Transduct Target therapy. 2017;2:1–9. 10.1038/sigtrans.2017.23 PMC5661633 29158945

[26] Gao H, Wang X, Qu X, Zhai J, Tao L, Zhang Y, Song Y, Zhang W. Omeprazole attenuates cisplatin-induced kidney injury through suppression of the TLR4/NF-κB/NLRP3 signaling pathway. Toxicology. 2020;440:152487. 32418911 10.1016/j.tox.2020.152487

