# Supplementary figures and images for: Comparison of the anti-inflammatory effects of esomeprazole and fexuprazan in lipopolysaccharide-stimulated RAW 264.7 macrophages
Source: BMC Pharmacol Toxicol. 2026 May 9;27:93. doi: 10.1186/s40360-026-01147-7 (PMC13326150; doi:10.1186/s40360-026-01147-7)

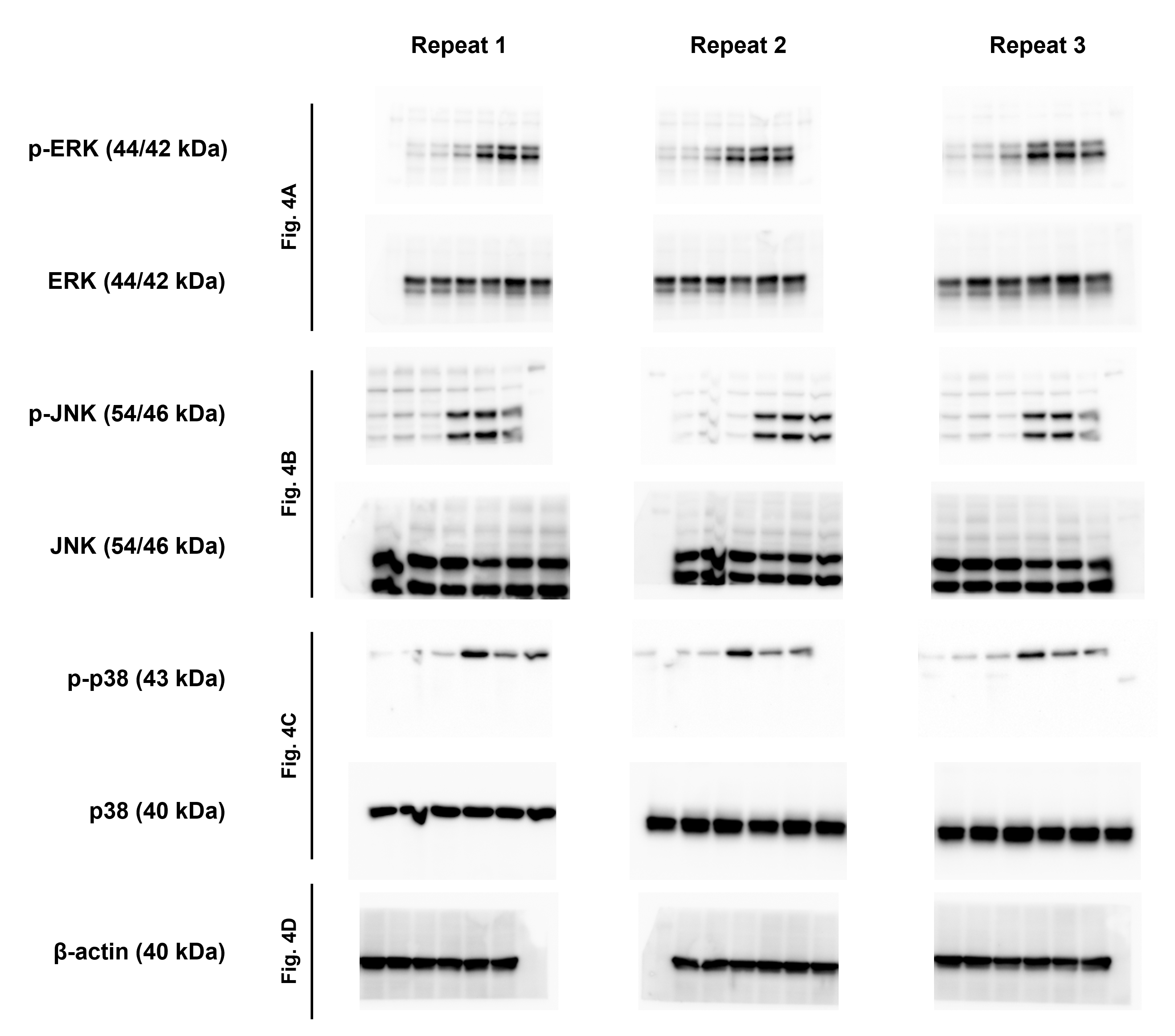

Supplement: Supplementary file 1 — Supplementary Material 1 [file 40360_2026_1147_MOESM1_ESM.tif]

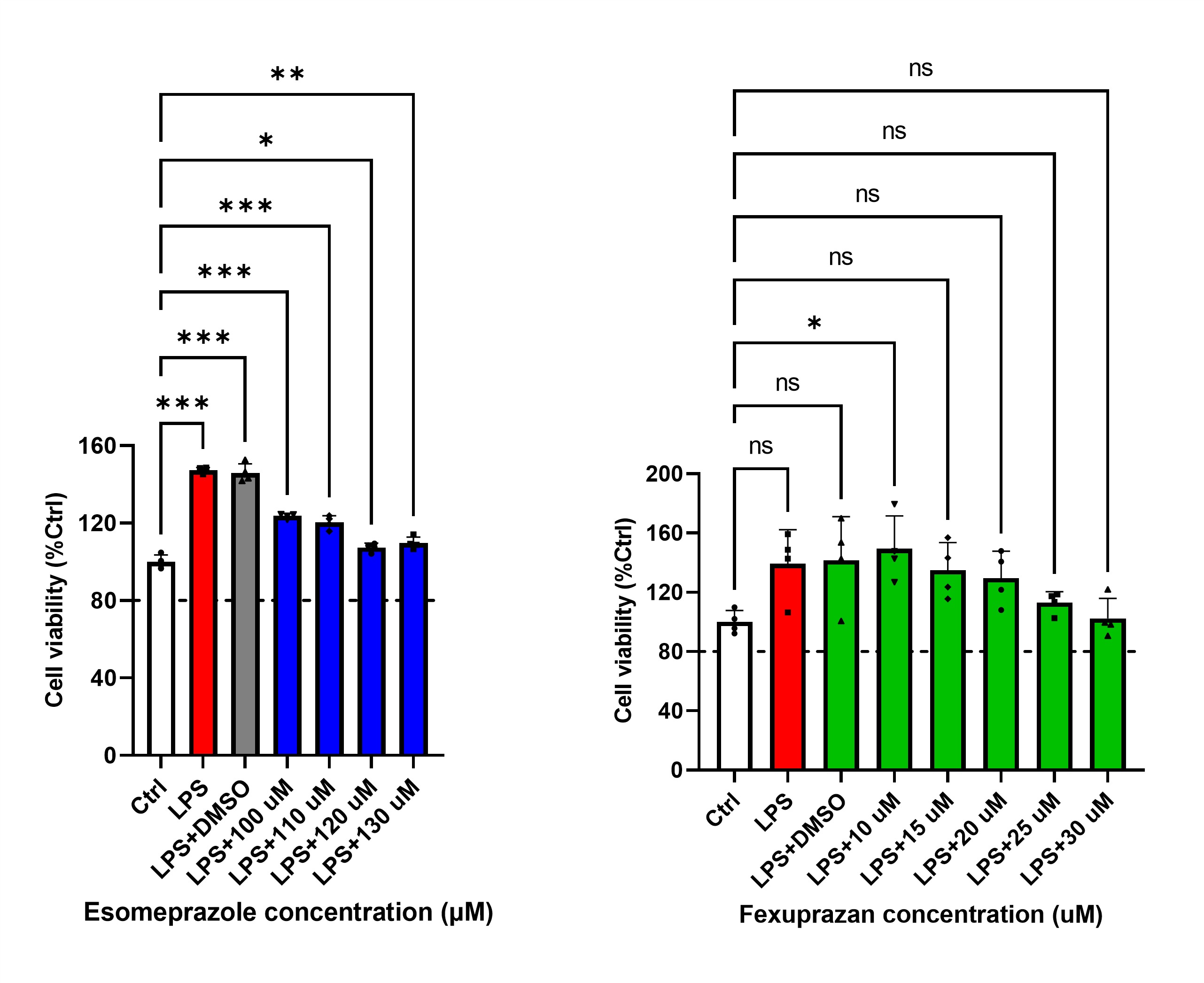

Supplement: Supplementary file 2 — Supplementary Material 2 [file 40360_2026_1147_MOESM2_ESM.tif]

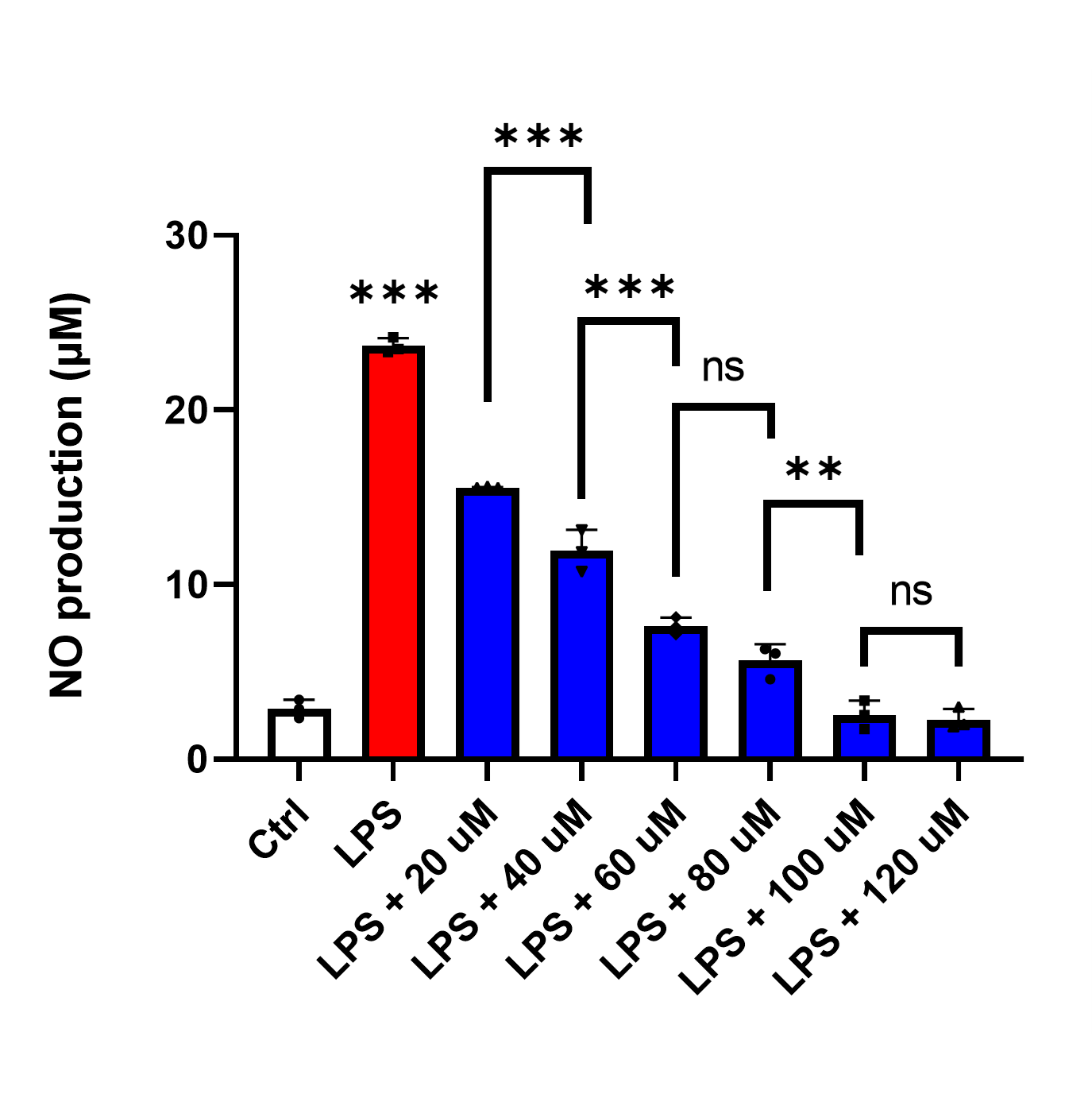

Supplement: Supplementary file 3 — Supplementary Material 3 [file 40360_2026_1147_MOESM3_ESM.tif]

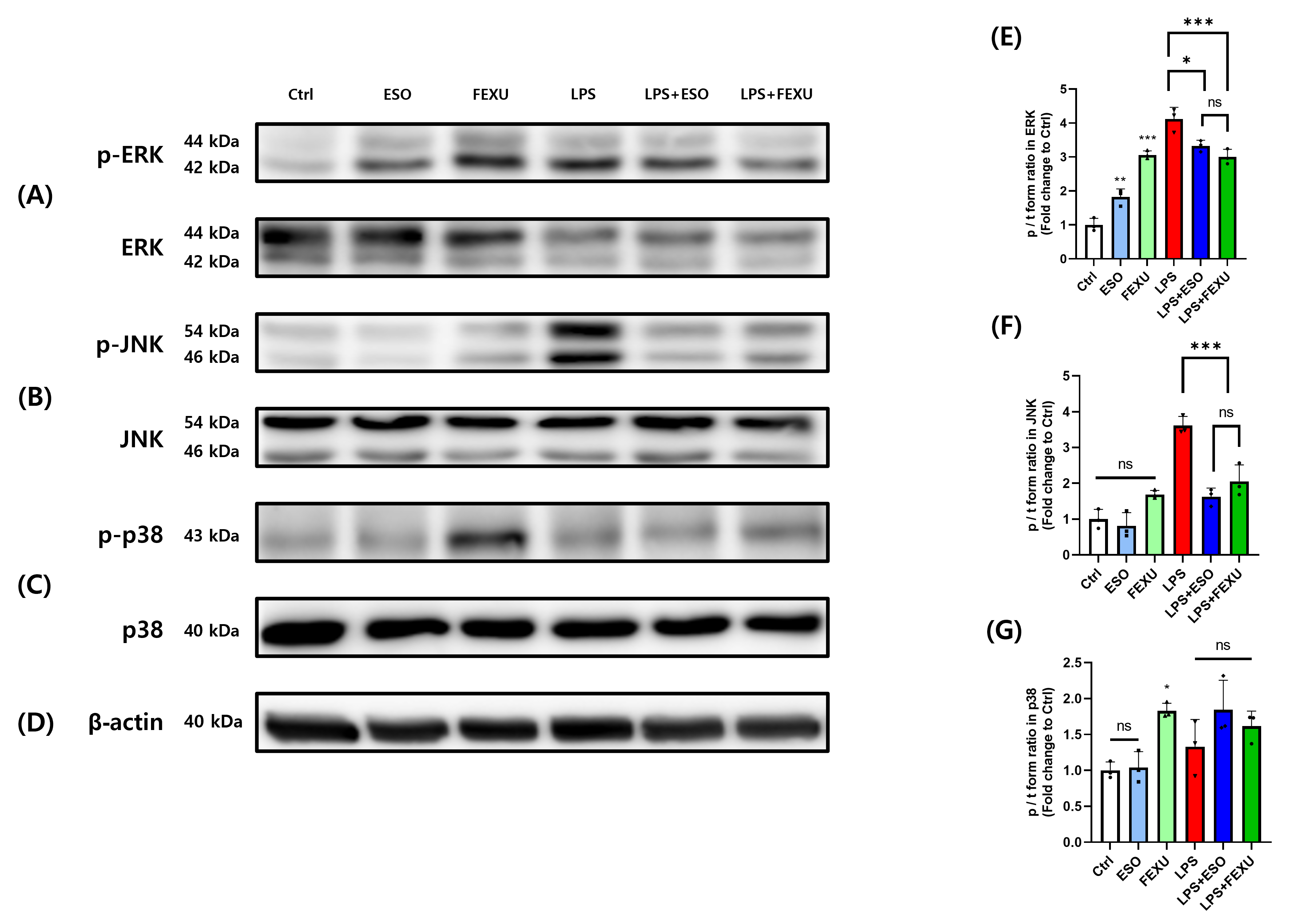

Supplement: Supplementary file 4 — Supplementary Material 4 [file 40360_2026_1147_MOESM4_ESM.tif]

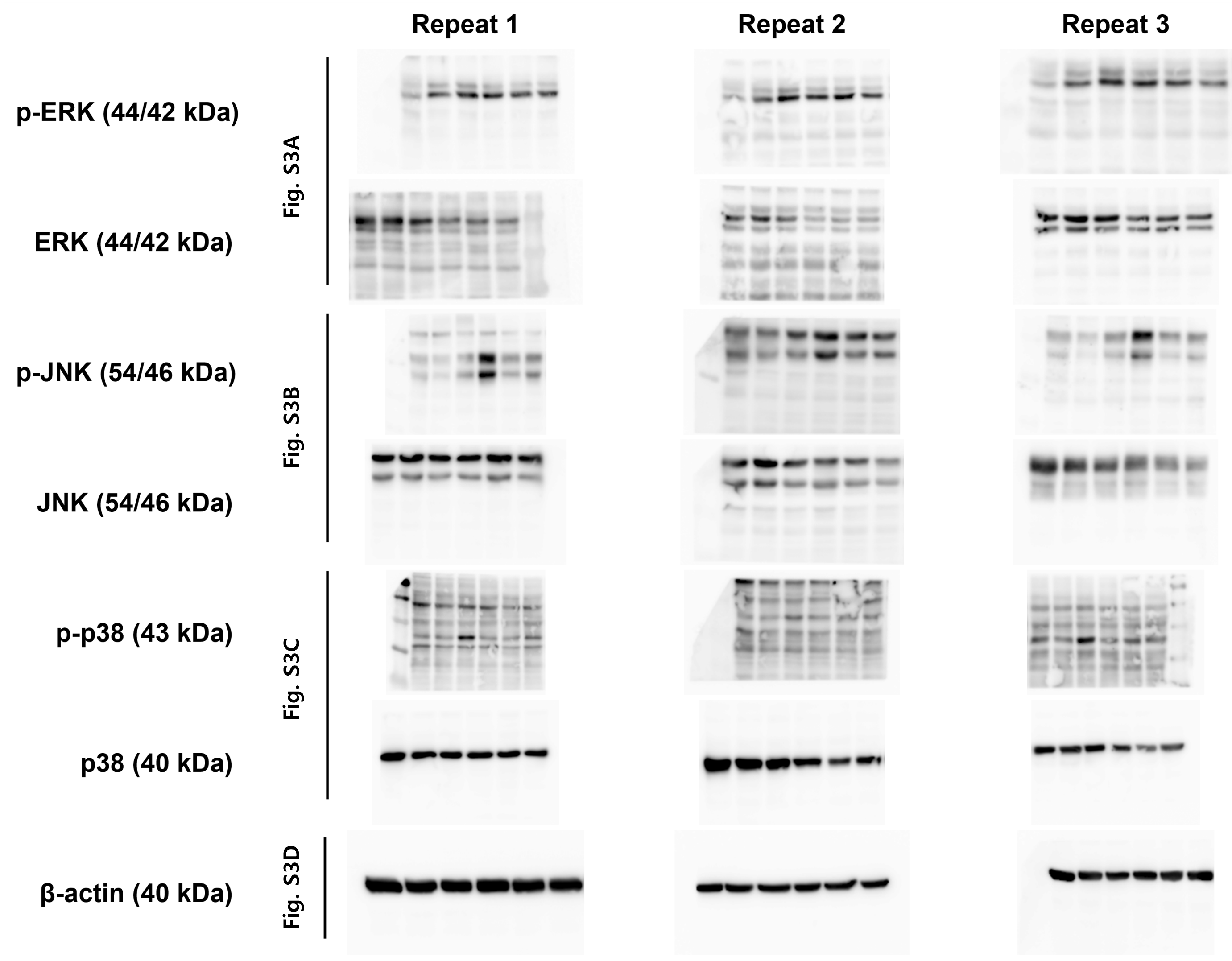

Supplement: Supplementary file 5 — Supplementary Material 5 [file 40360_2026_1147_MOESM5_ESM.tif]
