# Supplementary material for: Comparison of the anti-inflammatory effects of esomeprazole and fexuprazan in lipopolysaccharide-stimulated RAW 264.7 macrophages
Source: BMC Pharmacol Toxicol. 2026 May 9;27:93. doi: 10.1186/s40360-026-01147-7 (PMC13326150; doi:10.1186/s40360-026-01147-7)
Supplement: Supplementary file 6 — Supplementary Material 6 [file 40360_2026_1147_MOESM6_ESM.docx]

**Supplementary materials**

**Comparison of the anti-inflammatory effects of esomeprazole and fexuprazan in lipopolysaccharide-stimulated RAW 264.7 macrophages**

Gi-Beom Ju ^1†^, Seong Jun Kim ^1†^, Daye Lee ^1^, Min Je Kim ^1^, Wan-Kyu Ko ^2^, Min Jai Cho ^3*^, and Seil Sohn ^1, 4*^

^1^ Department of Life Science, CHA University, 335, Pangyo-ro, Bundang-gu, Seongnam-si 13488, Gyeonggi-do, Republic of Korea

^2^ Department of Chemistry and Chemical Biology, Rutgers, The State University of New Jersey, 123 Bevier Road, Piscataway, New Jersey 08854, USA

^3^ Department of Neurosurgery, Chungbuk National University College of Medicine, Chungbuk National University Hospital, 776, 1Sunhawn-ro, Seowon-gu, Cheongju-si 28644, Chungcheong-do, Republic of Korea

^4^ Department of Neurosurgery, CHA Bundang Medical Center, 59, Yatap-ro, Bundang-gu, Seongnam-si 13496, Gyeonggi-do, Republic of Korea

^†^Gi-Beom Ju and Seong Jun Kim contributed equally to this work.

^*^ Correspondence

Min Jai Cho

ulbo811211@naver.com

Seil Sohn

sisohn@cha.ac.kr

**
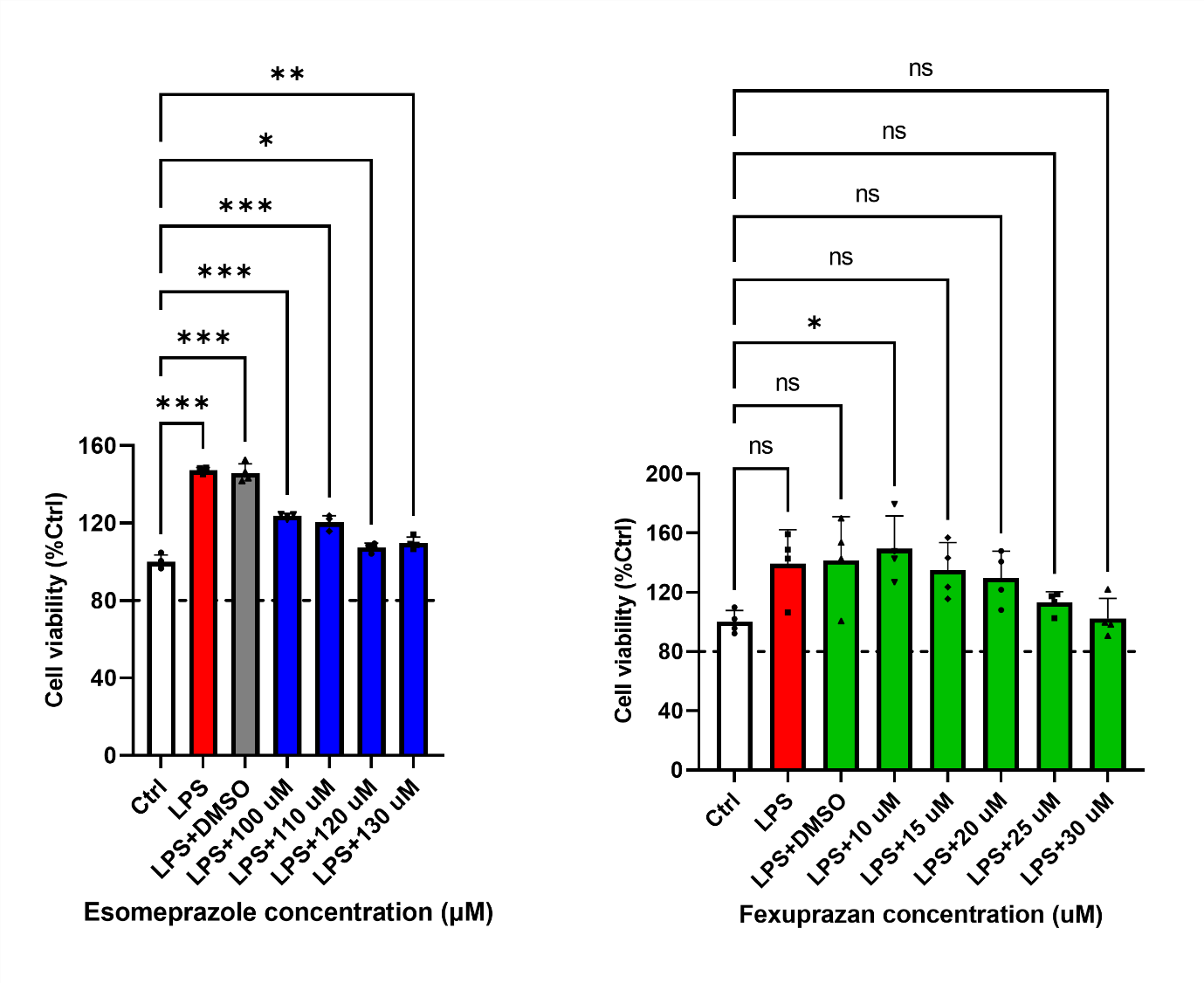
Fig. S1.** Cell viability of lipopolysaccharide (LPS, 1 μg/mL)-stimulated RAW 264.7 macrophages treated with ESO (100, 110, 120, and 130 μM) or FEXU (10, 15, 20, 25, and 30 μM) for 24 h. The results are presented as the mean ± standard deviation (SD, n = 4 per group), not significant (ns), * *p* < 0.05, and *** *p* < 0.001.


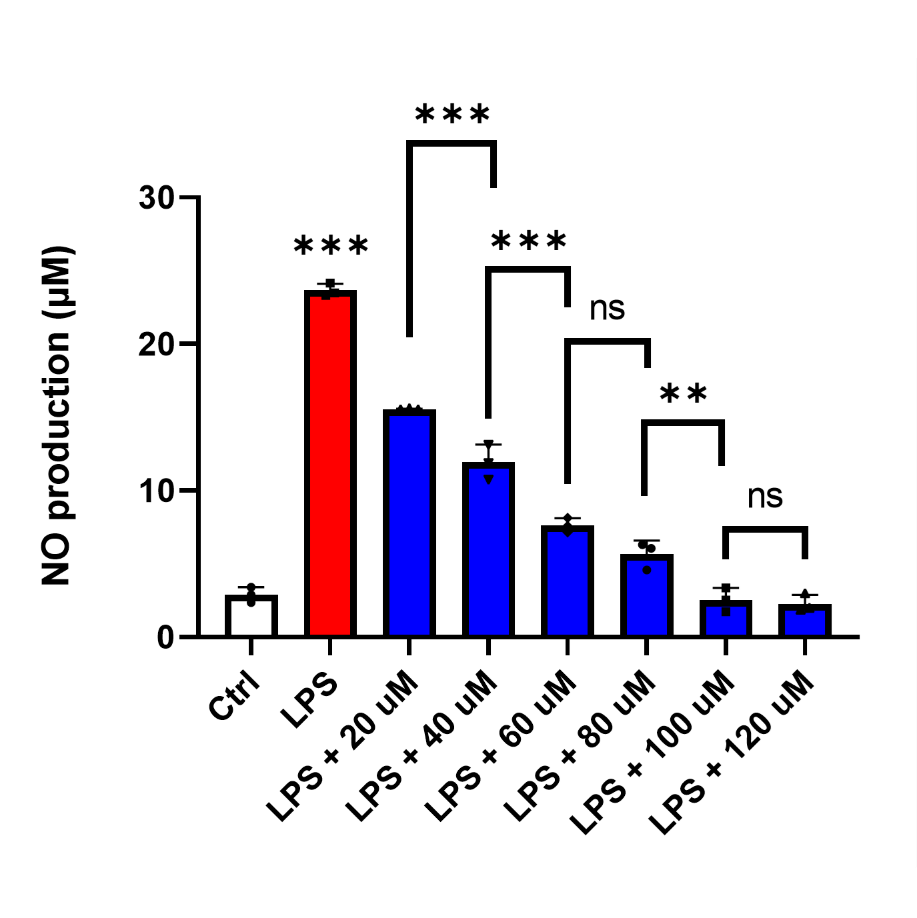


**Fig. S2.** Effect of lower concentrations of ESO on nitric oxide (NO) production in LPS-stimulated RAW 264.7 macrophages for 24 h. The results are presented as the mean ± SD (n = 3 per group), ns, ** *p* < 0.01, *** *p* < 0.001.

**
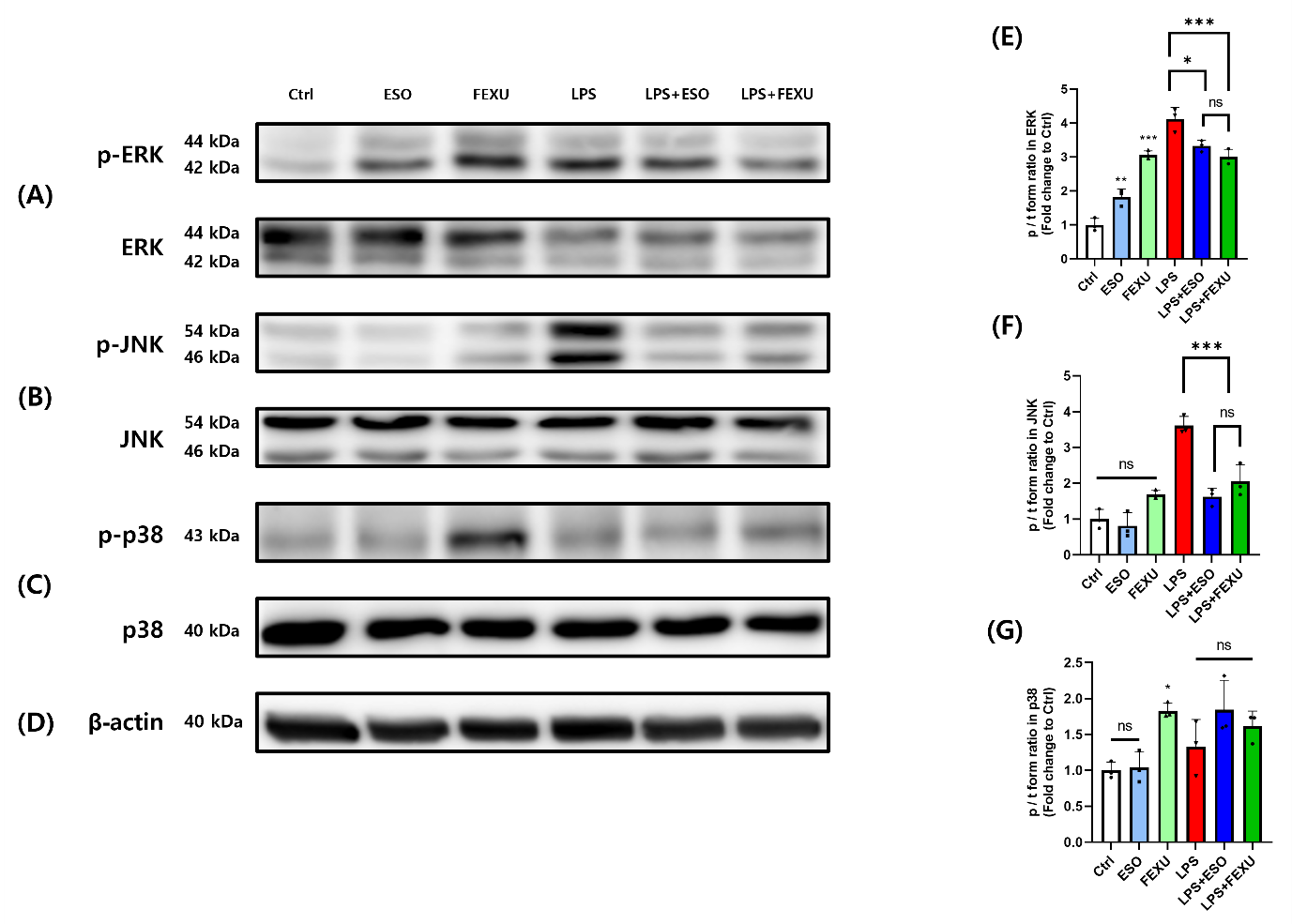
**

**Fig. S3.** The phosphorylation activities of the mitogen-activated protein kinase (MAPK) signaling pathway in LPS (1 μg/mL)-stimulated RAW 264.7 macrophages treated with ESO (120 μM) or FEXU (20 μM) after 24 h. Representative images of the p and t forms of extracellular signal-regulated kinase (ERK, A), c-Jun N-terminal kinase (JNK, B), p38 (C), and β-actin (D). Quantitative analysis of the p / t ratios of ERK (E), JNK (F), and p38 (G). The results are presented as the mean ± SD (n = 3 per group), ns, * *p* < 0.05, ** *p* < 0.01, and *** *p* < 0.001.
